# Effect of Annealing Holding Time on Microstructure, Interface Diffusion Behavior, and Deformation Behavior of Cu/Al Composite Foil After Secondary Micro-Rolling

**DOI:** 10.3390/ma18235418

**Published:** 2025-12-01

**Authors:** Xu Li, Hongmei Zhang, Jianling Wang, Guoao Yu, Zhengyi Jiang

**Affiliations:** 1 School of Materials and Metallurgy, University of Science and Technology Liaoning, Anshan 114051, China17640259934@163.com (G.Y.); 2 Ansteel Group Chaoyang Iron and Steel Co., Ltd., Chaoyang 122000, China; wangjianling_2006@163.com; 3 School of Mechanical, Materials, Mechatronic and Biomedical Engineering, University of Wollongong, Wollongong, NSW 2522, Australia

**Keywords:** micro-rolling, Cu/Al composite foil, holding time, microstructure, interface diffusion behavior, deformation behavior

## Abstract

In this study, the Cu/Al composite foil with a thickness of 0.04 mm was prepared by a combination of secondary micro-rolling and intermediate annealing process. The influence of different holding times (40, 60, and 80 min) at an annealing temperature of 400 °C on the microstructure, interfacial diffusion behavior, and deformation behavior of the secondary micro-rolled Cu/Al composite foil was systematically investigated using scanning electron microscopy with energy dispersive spectroscopy (SEM-EDS) and X-ray diffraction (XRD). The experimental results showed that as the holding time increased, the grain sizes of both Cu and Al in the first and second micro-rolled samples and the thickness of the interfacial diffusion layer were increased, meanwhile the interfacial intermetallic compounds were consistently identified as CuAl_2_, Cu_4_Al, and Cu_9_Al_4_ at different holding times. When the holding time reaches 80 min, (CuAl) appears in the secondary micro-rolled specimens. Furthermore, with the increase in holding time, the protrusion height of the edge profile and the degree of edge cracking in the secondary micro-rolled specimens increase, demonstrating that the edge deformation behavior of the material was significantly influenced by the holding time.

## 1. Introduction

In recent years, advancements in precision micro-mechanics and electronics, particularly in micro-system technology, micro-electromechanical systems (MEMSs), and medical engineering, have driven increased demand for miniaturized components [1,2,3]. Meanwhile, with the growth in production and consumption of composite materials, ultra-thin metal composites have found widespread application across automotive, electronics, medical, military, aerospace, robotics, and home appliance industries due to their thermal resistance, corrosion resistance, and wear resistance [4,5]. The combination of these factors has driven significant research and development efforts focused on optimizing the processing and performance characteristics of ultra-thin metal composites. These composite materials offer key advantages across various applications by balancing strength, lightweight design, and resistance to degradation in harsh environments.

Zhang et al. [6] investigated the influence of size effect on the mechanical properties and fracture behavior of pure copper specimens with thicknesses of 0.1, 0.2, and 0.3 mm that had undergone annealing treatment at different temperatures. With the increase in grain size and the decrease in sample thickness, the fracture mode changes from the mixed type to the sliding type. Wang et al. [7] investigated the microstructure, mechanical properties, and formability of Cu/Al composite foil during melt flow. The results show that the Cu/Al composite foil exhibits the best plasticity at an annealing temperature of 400 °C. When the plastic strain continues to increase from the thick zone to the thin zone, the intermetallic compound layer produced during the annealing process breaks. Wang et al. [8] systematically examined the effect of precipitation particles on grain size in cold-rolled aluminum–manganese alloy foil after annealing at temperatures ranging from 100 to 550 °C. It was indicated that when the annealing temperature was decreased from 550 °C to 400 °C, the recrystallized grain size in both samples was nearly identical and a slight increase was observed. A further decrease in the annealing temperature from 400 °C to 330 °C was found to result in a greater increase in grain size, with a more pronounced increase being exhibited by the thinner foil samples. Under identical annealing conditions, the grain size of Cu was further increased by the secondary micro-rolling process, while the growth rate of Al grains was reduced [9]. Meanwhile, the intermetallic compounds CuAl_2_, Cu_4_Al, and Cu_9_Al were formed in the ultra-thin copper/aluminum composite plates after secondary micro-rolling at 360 °C; at the elevated temperatures of 400 °C and 440 °C, the intermetallic compounds CuAl_2_, Cu_4_Al, Cu_9_Al, and CuAl were formed [10]. Chen et al. [11] analyzed the microstructural evolution and interfacial behavior of the ultra-thin composite plate, and it was observed that at temperatures between 350 and 500 °C, the grain size of both Cu and Al metals increased with rising annealing temperature, with a faster growth rate being exhibited on the Al side. During the diffusion process, the interfacial diffusion between copper and aluminum elements was initiated within the 350–400 °C range, leading to the bonding strength of the composite plate being enhanced. Furthermore, virtually no pore defects were exhibited by the interfacial diffusion layer. Jing et al. [12] systematically characterized the effects of grain size and reduction ratio on non-uniform deformation, edge cracking, and microstructure in pure copper foil, and it was observed that as grain size and reduction were increased, edge deformation was made more non-uniform, with more pronounced edge bulging and an increased extent of non-uniform expansion being induced. Edge cracking occurrence is influenced by the synergistic interaction of grain size and reduction ratio. Xu et al. [13] studied the size effects on the forming limit and fracture of pure copper foil with three different thickness-to-diameter ratios (T/D) of 100, 200, and 400 µm, and it was found that the forming limit was reduced with increasing T/D. Moreover, when the sheet thickness contained only one or two grains, the forming limit was significantly influenced by the behavior of a single grain As the (T/D) ratio increased, the fracture surface exhibited a reduction in the number of microvoids. Furthermore, microvoids were rarely observed when the sheet thickness contained only one or two grains. During the initial stage of plastic deformation, strain localization and non-uniform deformation became pronounced. Chen et al. [14] investigated the interfacial evolution and mechanical properties of ultra-thin copper–aluminum composite plates subjected to heat treatment within the 350–500 °C temperature range, and it was observed that a transition from mechanical bonding to metallurgical bonding was achieved at the interface, accompanied by a significant increase in bonding strength. Furthermore, an increase in the microhardness of the interface with rising annealing temperature was also recorded. Wang et al. [15] conducted further rolling of copper–aluminum composite strips with a cast-state interfacial layer thickness of 65 µm, and a composite strip with a total thickness of approximately 50 µm was successfully produced. Wang et al. [16] examined the growth mechanisms of intermetallic compounds and the evolution of microstructure and texture in copper and aluminum matrices, and the mechanical properties and formability of copper/aluminum composite foil were targeted for enhancement. Li et al. [17] conducted microstructural characterization and uniaxial tensile testing on Cu/Al layered composites to investigate interfacial effects and fracture mechanisms. Fracture behavior was evaluated using electron microscope images taken before and after tensile testing. Kim et al. [18] studied the intermetallic compound layer thickness and crack spacing in Cu/Al/Cu composite plates, and it was found that both were increased with rising heat treatment temperature. Furthermore, the correlation between peel strength and intermetallic compound layer thickness was supported when a thick, continuous intermetallic compound layer was formed after heat treatment at 400–500 °C. Fu et al. [19] investigated the brittle intermetallic compounds first consisting of Al_4_Cu_9_, AlCu_3_, and Al_2_Cu at the interfaces of Cu/Al/Cu composite plates after annealing. A decrease in the tensile strength of the composite was observed with increasing annealing temperature, while the elongation was found to first increase and then decrease beyond a critical point. This phenomenon was attributed to the evolution of intermetallic compounds at the composite interfaces. Zhao et al. [20] systematically studied the effect of the asynchronous speed ratio on the thickness ratio of copper to aluminum in composited strips using an asynchronous rolling process, and an increase in the Cu/Al thickness ratio with the increasing asynchronous speed ratio was observed. For micro-rolled Cu/Al composite foil, the microstructure, diffusion behavior at the composite interface, and deformation behavior are significantly influenced by size effects. The non-uniformity of the edge profile, particularly the occurrence of edge cracking, is a key factor limiting their performance and reliability. Therefore, this paper investigates the effect of annealing holding time on microstructure, interface diffusion behavior, and deformation behavior of Cu/Al composite foil after secondary micro-rolling and deeply explores its mechanism of action.

## 2. Materials and Methods

### 2.1. Experimental Materials

Ultra-thin T2 copper foil with a thickness of 0.1 mm and 1060 aluminum foil were selected as the materials for this experimental study. Their chemical compositions are shown in Table 1 and Table 2.

### 2.2. Experimental Methods

**Figure 1:**
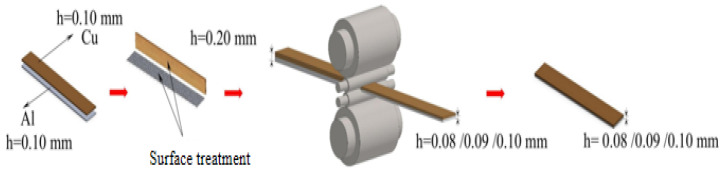
Experimental process flow chart of primary micro-rolling.

The 0.10 mm T2 Cu and 0.10 mm 1060 Al foil were selected as the experimental materials. The Cu and Al are uniformly ground along the rolling direction on the surface to be combined at a rotational speed of 8000 r/min using a small electric mill. This is more conducive to the mechanical interlocking between the T2 Cu extremely thin plate and the 1060 Al extremely thin plate interface during the micro-rolling process. The thickness of the Cu/Al composite foil after rolling is controlled by adjusting the downward force (800–900 N), with a rolling speed of 3 r/min. No lubricating reagent is used between the Cu and Al. Through first micro-rolling, Cu/Al composite foil was prepared. Subsequently, annealing heat treatment under different conditions was performed on the Cu/Al composite foil to obtain single-pass micro-rolled Cu/Al composite foil with varying grain sizes, as shown in Figure 1. The first micro-rolled Cu/Al composite foil with a moderate grain size, uniform diffusion layer, and appropriate thickness after annealing heat treatment was selected for secondary micro-rolling. This process yielded an even thinner secondary micro-rolled Cu/Al composite foil.

The Cu/Al composite foil, after a single micro-rolling pass, was subjected to an annealing heat treatment in a vacuum tube furnace. Three distinct annealing parameters were employed and designated as T1 to T3, as detailed in Table 3. The specifications of the annealing equipment are provided in Table 4.

**Figure 2:**
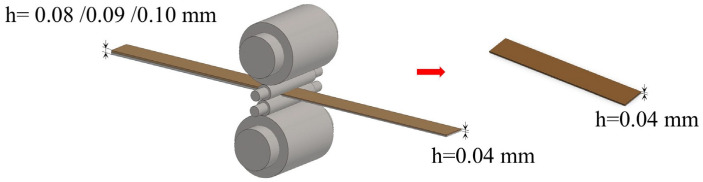
Experimental process flow chart of secondary micro-rolling.

Before conducting the secondary micro-rolling, the edges of the samples after the first micro-rolling annealing should be ground with 2000-mesh sandpaper to reduce the impact of edge unevenness on the experimental results. The rolling force was adjusted to evenly roll them to the target thickness of 0.04 mm. The specific procedure is illustrated in Figure 2: for samples with an annealed thickness of 0.10 mm, a 60% reduction rate was applied to achieve 0.04 mm; for annealed samples with thicknesses of 0.09 mm and 0.08 mm, reductions of 55.6% and 50%, respectively, were applied to achieve 0.04 mm. The rolling direction aligned with the micro-rolling direction. Subsequent annealing followed identical procedures to the first rolling process.

The effects of size effects on the microstructure and diffusion behavior at composite interfaces in micro-rolled Cu/Al foil are investigated. The first micro-rolled Cu/Al composite foil with an annealing temperature of 400 °C and a holding time of 80 min was subjected to a second micro-rolling process to produce a 0.04 mm thick sheet. Microstructural observations, interfacial element diffusion analysis, and X-ray diffraction (XRD) testing were conducted on the resulting sheet. Quantitative analysis was performed on the Cu and Al grain sizes and the thickness of the diffusion layer at the interface for micro-rolled Cu/Al composite foil with different holding times. Qualitative analysis was conducted on the intermetallic compounds in the diffusion layer at the interface.

The effects of size effects on the deformation behavior of micro-rolled Cu/Al composite foil is investigated. The first micro-rolled Cu/Al composite foil with an annealing temperature of 400 °C and a holding time of 80 min was subjected to secondary micro-rolling to produce a 0.04 mm secondary micro-rolled Cu/Al composite foil. The secondary micro-rolled Cu/Al composite foil was treated with different holding times. The edges were observed through an ultra-depth-of-field 3D profile microscope (VHX-5000, KEYENCE, Osaka, Japan) to explore the influence of microscopic grain size and macroscopic geometric size on the edge profile and edge cracking of micro-rolled Cu/Al composite foil.

## 3. Experimental Results

### 3.1. Effect of Annealing Holding Time on Microstructure of Cu/Al Composite Foil After Secondary Micro-Rolling

#### 3.1.1. Effect of Holding Time on Microstructure of Cu/Al Composite Foil Produced by First Micro-Rolling

**Figure 3:**
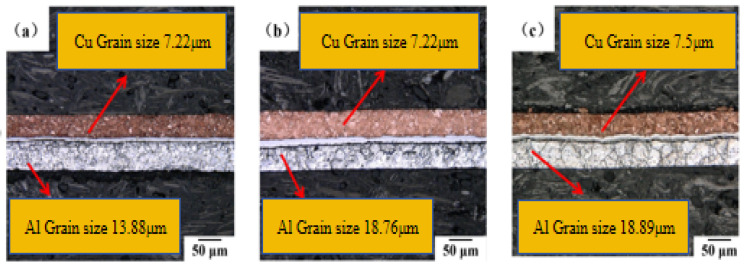
Microstructure of first micro-rolled ultra-thin Cu/Al composite foil at different holding times: (**a**) 40 min, (**b**) 60 min, (**c**) 80 min.

The microstructures of first micro-rolling Cu/Al composite foil at different holding times are shown in Figure 3a–c. The wire cutting method was adopted for determination. When the annealing temperature was 400 °C and the holding time was 40 min, in the thickness direction, the Cu grains are 5–6 layers and the Al grains are 4–5 layers. When holding for 60 min, in the thickness direction, there are 3 to 4 layers of Cu grains and 2 to 3 layers of Al grains. When holding for 80 min, in the thickness direction, the Cu grains are 3–4 layers and the Al grains are 2–3 layers.

**Figure 4:**
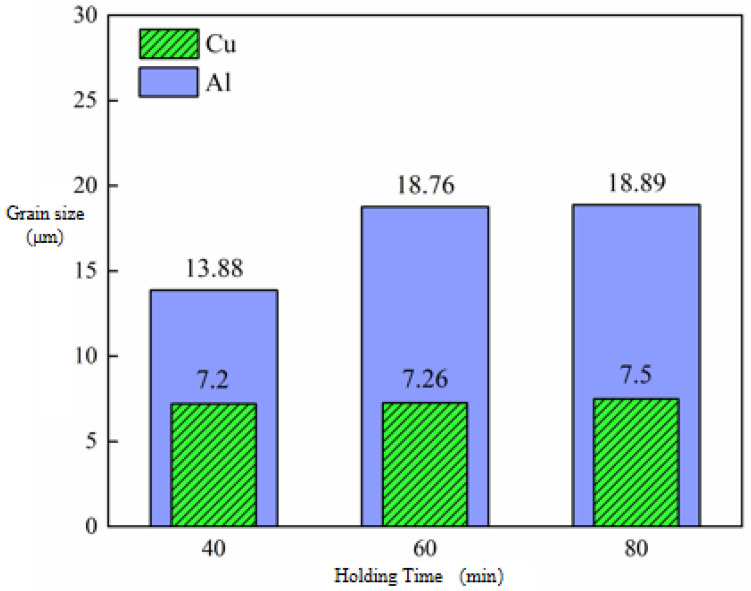
Grain size change in Cu and Al of first micro-rolled ultra-thin Cu/Al composite foil at different holding times.

The grain size changes in Cu and Al after the first micro-rolling at different holding times are shown in Figure 4. The grain sizes of Cu and Al increase with the increase in holding time. This is because the longer holding time provides sufficient time for grain boundary migration and coalescence.

At an annealing temperature of 400 °C, when the holding time was extended from 40 min to 80 min, the Cu grain size gradually increased from 7.22 μm to 7.5 μm, while the Al grain size grew from 13.88 μm to 18.89 μm. This indicates that as the holding time increases, the grain coarsening of Al becomes more pronounced. During shorter holding times, the deformation energy storage is rapidly consumed through recrystallization nucleation and grain growth in the Al matrix, leading to a rapid increase in grain size. A significant decrease in the Al grain coarsening rate is observed when the holding time exceeds 60 min, which is attributed to the grain size approaching thermodynamic equilibrium [21]. Conversely, recrystallization is completed by Cu grains within 40 min, with only limited grain boundary migration being induced thereafter.

#### 3.1.2. Effect of Holding Time on Microstructure of Secondary Micro-Rolled Cu/Al Composite Foil

**Figure 5:**
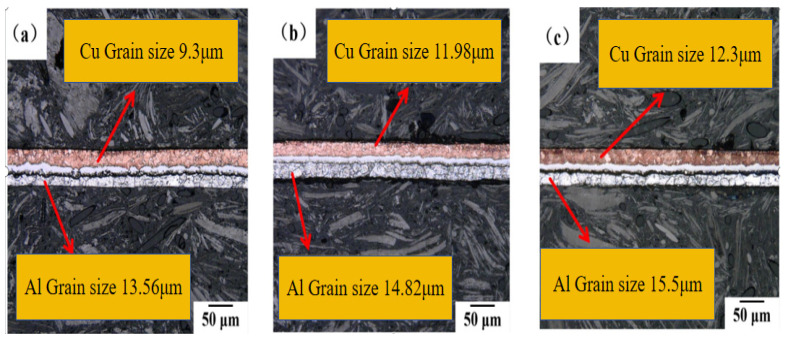
Microstructure of secondary micro-rolled ultra-thin Cu/Al composite foil at different holding times: (**a**) 40 min, (**b**) 60 min, (**c**) 80 min.

The microstructures of Cu/Al composite foil after secondary micro-rolling at different holding times are shown in Figure 5a–c. The wire cutting method was adopted for determination. When the annealing temperature is 400 °C and the holding time is 40 min, in the thickness direction, the Cu grains are 1 to 3 layers and the Al grains are 1 to 2 layers. When the holding time is 60 min, in the thickness direction, the Cu grains are 1 to 3 layers and the Al grains are 1 to 2 layers. When the holding time is 80 min, in the thickness direction, there are 1 to 2 layers of Cu grains and 1 to 2 layers of Al grains.

**Figure 6:**
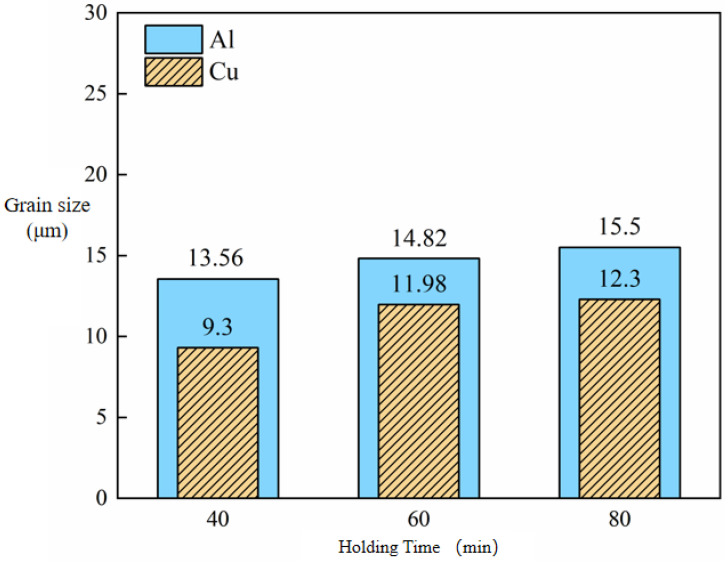
Grain size change in Cu and Al of secondary micro-rolled ultra-thin Cu/Al composite foil at different holding times.

The grain size changes in Cu and Al after secondary micro-rolling at different holding times are shown in Figure 6. It can be observed that both Cu and Al grain sizes increase with extended holding time. At the annealing temperature of 400°C, when the holding time was extended from 40 min to 80 min, the Cu grain size increased from 9.3 μm to 12.3 μm, while the Al grain size increased from 13.56 μm to 15.5 μm. At the same holding time, compared with the first micro-rolling, the coarsening rate of Cu grains in the second micro-rolling is significantly higher. Meanwhile, the size of Al grains still shows an increasing trend with the increase in holding time, but the increase is significantly lower than that after annealing in the first micro-rolling.

### 3.2. Effect of Annealing Holding Time on Diffusion Behavior at the Composite Interface of Cu/Al Composite Foil After Secondary Micro-Rolling

#### 3.2.1. Effect of Holding Time on Interfacial Diffusion Behavior in First Micro-Rolled Cu/Al Composite Foil

**Figure 7:**
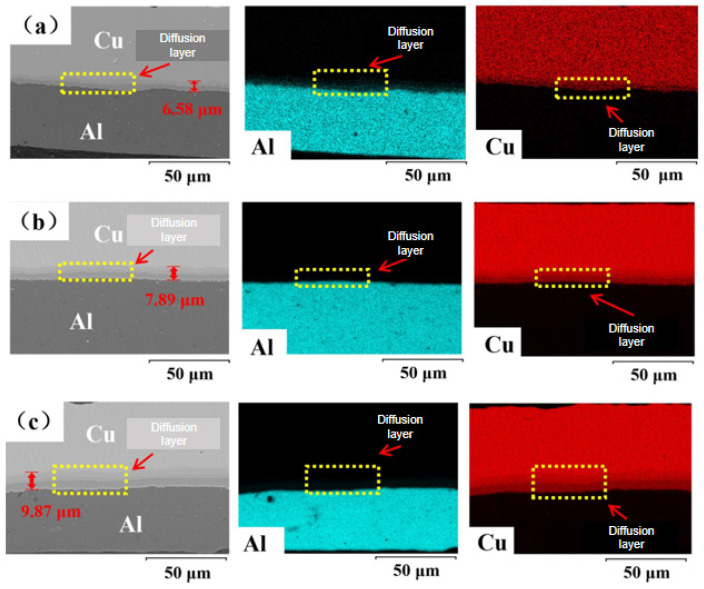
Interface element diffusion of first micro-rolled ultra-thin Cu/Al composite foil at different holding times: (**a**) 40 min, (**b**) 60 min, (**c**) 80 min [9].

**Figure 8:**
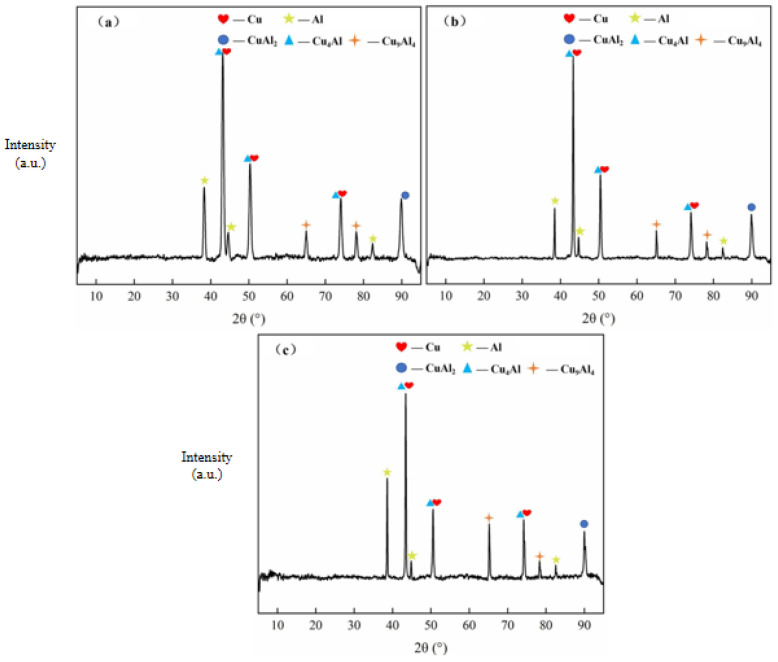
X-ray diffraction pattern of first micro-rolled ultra-thin Cu/Al composite foil at different holding times: (**a**) 40 min, (**b**) 60 min, (**c**) 80 min.

The diffusion behavior of interfacial elements in Cu/Al composite foil after the first micro-rolling process at different holding temperatures is shown in Figure 7a–c. Test results indicate that at an annealing temperature of 400°C, the interfacial diffusion layer thickness was 6.58 μm for a holding time of 40 min, 7.89 μm for 60 min, and 9.87 μm for 80 min. The interfacial diffusion layer remained continuous across all holding times. X-ray diffraction patterns of Cu/Al composite foil after the first micro-rolling process at different holding times are shown in Figure 8a–c. The interfacial intermetallic compounds were consistently identified as CuAl_2_, Cu_4_Al, and Cu_9_Al_4_ at different holding times.

Analysis of the two diagrams reveals that as the holding time increases, both Cu and Al grain sizes grow, while the thickness of the interfacial diffusion layer also increases. This indicates that the extension of the holding time provides more time for the mutual diffusion of Cu and Al atoms at the interface, thereby promoting the increase in the thickness of the diffusion layer. At different holding times, the interfacial diffusion layer is continuous, indicating that when the annealing temperature reaches 400 °C, Cu and Al atoms can fully diffuse into each other. The holding time mainly affects the diffusion process but does not change the types of intermetallic compounds. This means that the formation of these intermetallic compounds is mainly restricted by atomic diffusion kinetics. Under the conditions of this experiment, the formation process is basically completed. If the holding time is further extended, no new intermetallic compounds will be produced.

#### 3.2.2. Effect of Holding Time on Interfacial Diffusion Behavior in Secondary Micro-Rolled Cu/Al Composite Foil

**Figure 9:**
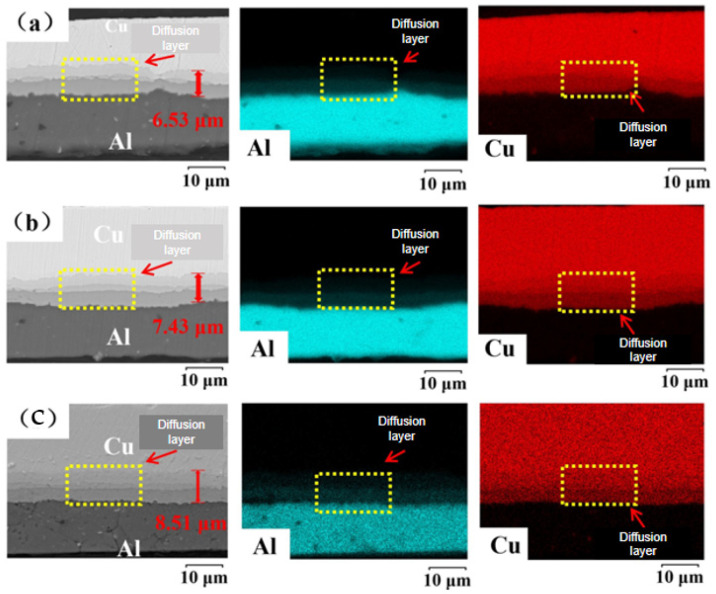
Interface element diffusion of secondary micro-rolled ultra-thin Cu/Al composite foil at different holding times: (**a**) 40 min, (**b**) 60 min, (**c**) 80 min.

**Figure 10:**
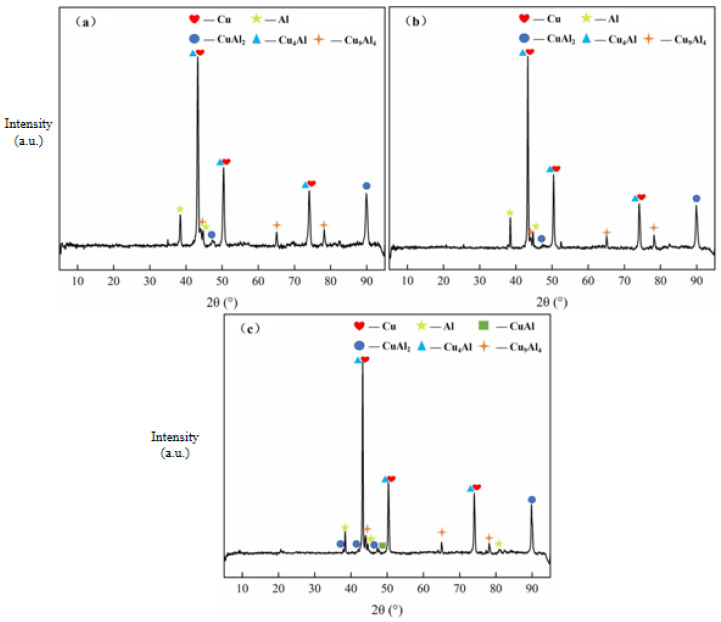
X-ray diffraction pattern of secondary micro-rolled ultra-thin Cu/Al composite foil at different holding times: (**a**) 40 min, (**b**) 60 min, (**c**) 80 min.

The interfacial element diffusion behavior in Cu/Al composite foil after secondary micro-rolling at different holding times is shown in Figure 9a–c. According to the test results, at an annealing temperature of 400 °C, when the holding time was 40 min, the interfacial diffusion layer thickness was 6.53 μm; when the holding time was 60 min, the interfacial diffusion layer thickness was 7.43 μm; and the interfacial diffusion layer thickness was 8.51 μm at 80 min. The diffusion layer remained continuous across all holding times. The X-ray diffraction patterns of the secondary micro-rolled Cu/Al composite foil at different holding times are shown in Figure 10a–c. It can be observed that at an annealing temperature of 400 °C and holding times of 40 min and 60 min, the intermetallic compounds present are CuAl_2_, Cu_4_Al, and Cu_9_Al_4_. Compared to the same annealing conditions after first rolling, the types and quantities of intermetallic compounds remain consistent. When the holding time is increased to 80 min, a new intermetallic compound, CuAl, appears in addition to CuAl_2_, Cu_4_Al, and Cu_9_Al_4_.

Analysis of the two diagrams reveals that as the holding time increases, both Cu and Al grain sizes grow, while the thickness of the interfacial diffusion layer also increases. Similarly to the effect of annealing temperature, atomic diffusion at the interface is promoted by extending the holding time, though the diffusion rate is observed to be relatively slow. It is suggested that the influence exerted by the holding time is less pronounced than that of the annealing temperature. At the same holding time, compared with the first micro-rolling, the thickness of the interfacial diffusion layer in the second micro-rolling is slightly reduced, making it less likely to pass through the potential barrier of intermetallic compounds on the interface. X-ray diffraction analysis revealed that at an annealing temperature of 400 °C with holding times of 40 min and 60 min, only three intermetallic compounds—CuAl_2_, Cu_4_Al, and Cu_9_Al_4_—were detected at the interface, similar to the results after first micro-rolling. However, when the holding time was extended to 80 min, a new intermetallic compound, CuAl, appeared. Secondary micro-rolling further reduces the material thickness and introduces more interfacial strain and defects. This reduces the activation energy of atomic diffusion and promotes the formation of more stable intermetallic compounds such as CuAl. After the second micro-rolling, the material became thinner and the grains coarser, which increased the chemical potential gradient in the interface region and accelerated the diffusion of copper atoms. Extending the holding time (80 min) provides sufficient time for atoms to overcome the energy barrier, allowing the CuAl phase to form. The increase in holding time provides more time for Cu and Al atoms to overcome the energy barrier for the formation of CuAl, promoting the reaction of other intermediate phases converting into CuAl. Therefore, the types and quantities of intermetallic compounds at the interface can be selectively regulated through careful control of the annealing temperature and holding time, thereby enabling the performance of the composite material to be optimized.

### 3.3. Effect of Annealing Holding Time on Edge Deformation Behavior of Cu/Al Composite Foil After Secondary Micro-Rolling

#### 3.3.1. Effect of Holding Time on Edge Profile of Secondary Micro-Rolled Cu/Al Composite Foil

**Figure 11:**
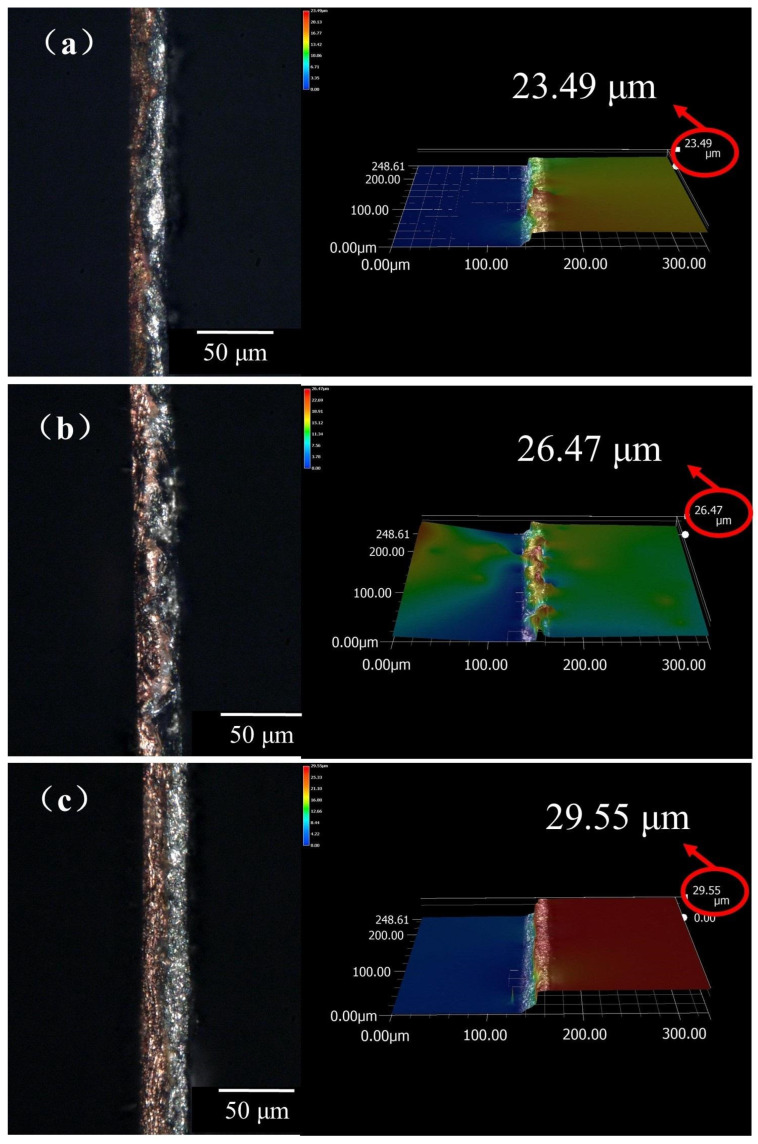
Edge profile of secondary micro-rolled ultra-thin Cu/Al composite foil at different holding times: (**a**) 40 min, (**b**) 60 min, (**c**) 80 min.

The edge profiles of Cu/Al composite foil after secondary micro-rolling at different holding times are shown in Figure 11 a–c. When the holding time at 400 °C and annealing duration were both 40 min, the protrusion height of the edge profile reached 23.49 μm. At an annealing time of 60 min, the protrusion height of the edge profile was 26.47 μm; at an annealing time of 80 min, the protrusion height reached 29.55 μm.

With increasing holding time, the protrusion height of the edge profile in the Cu/Al composite foil gradually rises, though the increase is limited. Combining the analysis of microstructure and interfacial diffusion behavior in the Cu/Al composite foil subjected to secondary micro-rolling with holding time, under annealing conditions at 400 °C, as the holding time extended from 40 min to 80 min, Cu grains grew from 9.3 μm to 12.3 μm, while Al grains increased from 13.56 μm to 15.5 μm. The number of grain layers in the thickness direction decreased to 1–2 layers, reducing the grains’ coordinated deformation capability and exacerbating edge profile irregularities. Additionally, the interfacial diffusion layer thickness increased from 6.53 μm to 8.51 μm, accompanied by elevated intermetallic compound content and increased brittleness. The material’s resistance to plastic deformation during subsequent rolling processes was further diminished, and the formation of edge profile protrusions was accelerated. Although the magnitude of changes induced by the extended holding time was limited, the impact on edge profiles was demonstrated, showing how macroscopic geometric characteristics are governed by microstructural evolution.

When the total thickness of the composite plate is reduced to 0.04 mm, half the material thickness is approached or even exceeded by the grain size on the Al side, and the ratio of grain size to specimen geometry is increased, resulting in the influence of individual grain behavior on the overall deformation being made more pronounced. With the holding time extended, Al grains are further coarsened and the number of grain layers is reduced, leading to the deformation coordination between grains being diminished. As a result, the edge regions are made more susceptible to stress concentration and non-uniform flow during rolling, which is manifested as an increase in the protrusion height of the edge profile.

#### 3.3.2. Effect of Holding Time on Edge Cracking in Secondary Micro-Rolled Cu/Al Composite Foil

**Figure 12:**
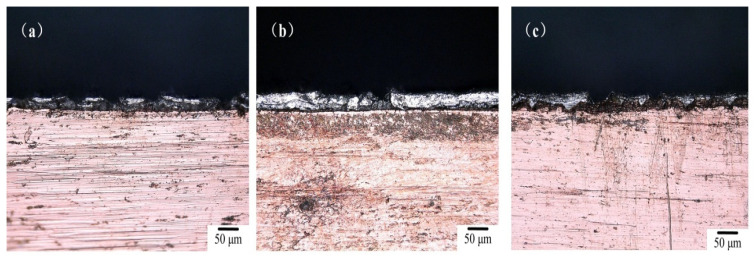
Edge profile of secondary micro-rolled ultra-thin Cu/Al composite foil at different holding times: (**a**) 40 min, (**b**) 60 min, (**c**) 80 min.

The edge cracks of the Cu/Al composite foil after secondary micro-rolling at different holding times are shown in Figure 12 a–c. It can be observed that as the holding time increases, the edge cracking intensifies.

Based on the analysis of the influence of holding time on the microstructure and interfacial diffusion behavior of secondary micro-rolled Cu/Al composite foil, it is observed that the grain sizes of both Cu and Al are increased by extended holding time. Particularly, the number of Al grain layers in the thickness direction is reduced, and the grain coordination deformation capability is diminished. During the secondary micro-rolling process, due to the size effect, local deformation is dominated by the behavior of a single or a few grains, which easily leads to the localization of edge strain.

With the holding time extended, the thickness of the interfacial diffusion layer is gradually increased, and the quantity and size of intermetallic compounds (such as CuAl_2_ and Cu_9_Al_4_) are also increased. These intermetallic compounds are characterized by high hardness and brittleness, and they are prone to becoming nucleation sites for microcracks during rolling deformation. Especially in edge regions with stress concentration, the presence of brittle phases is found to aggravate the initiation and propagation of cracks.

However, the formation and growth rates of intermetallic compounds are relatively slow, and their structures are maintained relatively stable within the experimental time scale. Therefore, although the interfacial reaction and the formation of brittle phases are promoted by extended holding time, the promotion of edge cracking is not drastic. The variation in edge cracking behavior is more significantly affected by the ratio of grain size to geometric dimension and is constrained by the deformation coordination between grains. When the grain size is increased and the number of grain layers is reduced, the plastic flow in the edge regions is made more non-uniform and local strain concentration is intensified, which results in a slight increase in the degree of edge cracking with prolonged holding time.

## 4. Conclusions

The effects of annealing holding time on the microstructure, composite interface diffusion behavior, and deformation behavior of Cu/Al composite foil after secondary micro-rolling were investigated. The findings are summarized as follows:

1. As holding time increases, both Cu and Al grain sizes in first and second micro-rolled specimens gradually increase; holding time has a more pronounced effect on the coarsening of the first rolled Al grains. Concurrently, the reduction in the number of Cu and Al grain layers reduces the grains’ ability to undergo coordinated deformation. Meanwhile, the coarsening of Cu grains after secondary micro-rolling significantly intensifies with increasing holding time.
2. As holding time increases, the atomic diffusion at the Cu/Al interface of the first and second micro-rolled specimens is promoted, resulting in a continuous increase in the thickness of the interfacial diffusion layer. XRD analysis indicated that the intermetallic compounds formed at different holding times contained CuAl_2_, Cu_4_Al, and Cu_9_Al_4_. When the holding time reaches 80 min, CuAl is generated in the secondary micro-rolled sample.
3. As holding time increases, the coordinated deformation ability of grains decreases, the content of brittle compounds between metals increases, and the plastic flow of the material in the edge area is uneven; the protruding height of the edge contour of the Cu/Al composite foil gradually increases after secondary micro-rolling, the degree of edge cracking slightly intensifies.

## Figures and Tables

**Table 1:** Chemical constituents of ultra-thin 1060 Al sheet, wt.%.

Al	Zn	V	Mn	Mg	Ti	Si	Cu	Fe
99.60	0.05	0.05	0.03	0.03	0.03	0.25	0.05	0.35

**Table 2:** Chemical constituents of ultra-thin T2 Cu sheet, wt.%.

Cu	Sb	Bi	As	Ni	Fe	Pb	P	S	Sn	Zn
99.90	<0.0010	<0.0004	<0.0010	0.0013	0.0074	0.0037	0.0017	0.0015	0.0023	0.0099

**Table 3:** Table of process parameters of annealing heat treatment.

Annealing Temperature °C	Heating Rate, °C/min	Holding Time, min	Annealing Condition Number
400	5	40	T1
400		60	T2
400		80	T3

**Table 4:** Parameter table of vacuum tube annealing furnace. (GR.TF60, Shguier, Shanghai, China).

Equipment Model	Equipment Parameters	Technical Indicators
GR.TF60-18	Rated voltage, V	380
	Rated Power, KW	7
	Maximum temperature, °C	1800
	Furnace chamber dimensions, mm	Φ 60~1000
	Common temperature, °C	1700

## Data Availability

The original contributions presented in this study are included in the article. Further inquiries can be directed to the corresponding authors.
